# 児童作文における文節数および係り受け距離の分布: 自然言語の特性と言語発達に伴う変異

**DOI:** 10.12688/f1000research.132383.1

**Published:** 2023-04-11

**Authors:** 今田水穂 今田

**Affiliations:** 1 Institute of Humanities and Social Sciences, University of Tsukuba, Tsukuba, Ibaraki, 305-8577, Japan

**Keywords:** sentence length, dependency distance, children's compositions, probability distribution, generalized linear mixed model, 文長, 係り受け距離, 児童作文, 確率分布, 一般化線形混合モデル

## Abstract

日本語の文における文節数および係り受け距離平均（MDD）の分布について、ランダムデータと児童作文データの比較、および学年による分布の変化を分析した。その結果、ランダムデータにおいては文節数は幾何分布、MDDは対数正規分布によく当てはまるのに対して、作文データにおいては文節数は学年によって対数正規分布からガンマ分布に推移し、MDDはガンマ分布によく当てはまることが分かった。また、MDDの平均はランダムデータでは文節数の対数に対して指数的に増大するのに対し、作文データでは線形に増大することが分かった。これは自然言語において係り受け距離が最適化されるという従来の知見を概ね支持する結果と言える。一方、MDDは学年に対して非単調な変化を示し、児童の言語発達の複雑さを示唆する。

## Abstract (en)

This study analyzed the distribution of the sentence length and mean of dependency distances (MDD) in Japanese sentences, comparing data from random sources with that obtained from children's compositions, and identifying changes in distribution according to grade level. Findings reveal that the sentence length in random data is well suited to a geometric distribution, whereas MDD is well suited to a lognormal distribution. In contrast, data from children's compositions show a shift in the distribution of the number of clauses from a lognormal to a gamma distribution, depending on the school year, with MDD suiting a gamma distribution. Mean MDD increases exponentially with the logarithm of the number of clauses in random data, while it increases linearly in composition data, thus generally supporting previous findings that dependency distances are optimized in natural language. However, MDDs exhibit non-monotonic changes with grades, suggesting the complexity of children's language development.

## 1. はじめに

本稿では、日本語の文における文長（文節数）および係り受け距離の分布の特性について検討し、児童作文における分布の変異を分析する。文長は文の複雑性を代表する基礎的な指標と見なされており、文の可読性（リーダビリティ）を評価するためにしばしば利用される（ 建石ほか 1988、 柴崎・原 2010、 李 2016）。文長は文字、形態素、文節などを単位として測定されるが、おおむね右に裾野が長い分布を示すことが知られており、文字数については対数正規分布ないしガンマ分布（ 安本 1958、 佐々木 1976、 新井 2001）、形態素数についてはハイパーパスカル分布 ( Ishida and Ishida 2007)、文節数については負の二項分布（ 古橋・早川 2012）の当てはまりがよいことが報告されている。

一方、同じ長さの文であってもその統語的な構造は多様であり、それによって統語的な複雑性に違いがある。よく知られた指標としては文の構成要素間の依存関係の距離 (dependency distance) があり、一般に距離の長い依存関係は認知的負荷が大きいため、自然言語では長距離の依存関係は避けられる傾向があると考えられている ( Gibson 1998)。依存距離の研究の多くは単語レベルの依存構造に依拠して行われており、その中には日本語を含む多言語における依存距離最小化の分析 ( Liu 2008、 Futrell *et al.* 2015、 Ferrer-i-Cancho *et al.* 2022) や、日本語における文章ジャンルの位相差の分析（ 李ほか 2022）が含まれる。一方で、日本語の統語構造の表現としては文節単位の係り受け構造が広く用いられてきた経緯があり、係り受け距離の分布が Zipf 則に近いこと ( Maruyama and Ogino 1993、 金 1996)、日本語学習者の係り受け距離平均について学習者のレベルに応じた増大が確認できないこと ( Komori *et al.* 2018) などが報告されている。日本語の構文情報についてはUD-Japanese BCCWJ（ 大村・浅原 2018）など単語単位の依存情報つきコーパスも開発されているものの、現在でも BCCWJ-DepPara（ 浅原・松本 2018）などの文節係り受け情報コーパスやCaboCha（ 工藤・松本 2002）などの文節係り受け解析器がよく用いられており、この構造の数学的特性を理解することは依然として有用性が高い。

そこで本稿では、文節数に基づく文長の分布、および一文あたりの係り受け距離平均の分布という 2 つの観点から、文の統語的構造の数学的特性を検討する。検討のために、2 種類のデータを用いる。ひとつはランダム性を持つ文節列および係り受け構造であり、もう 1 つは児童作文における文節係り受け構造である。これらのデータを分析対象とすることには、いくつかの意味がある。第 1 に、ランダムデータと作文データを比較することで、自然言語に固有の数学的特性を知ることができる。第 2 に、作文データを分析対象とすることで、児童の言語発達と統語的複雑性の関連を調べることができる。分布の特性は自然言語の特性の研究においては強い関心が置かれている一方で、可読性や言語発達の研究ではそれほど関心が置かれていない。可読性研究の多くは平均文長を説明変数として利用しているが、文長の分布は特に問題とされていない。 Komori *et al. *(2018) は日本語学習者のレベルと係り受けの距離や深さとの関連を調べているが、分布の正規性や等分散性を問題としない Brunner-Munzel 検定を使用している。 今田 (2021) ^1^ は児童の学年と係り受けの距離や深さとの関連を調べているが、ランダムデータに見られる対数正規分布をそのまま作文データに対して適用しており、自然言語に固有の特性を十分に考慮していない。本稿は、ランダムデータと作文データにおける文長と係り受け距離の分布の特性を詳細に検討し、その特性に基づいて児童の言語発達と統語的複雑性の関係を分析する。

## 2. 方法

### 2.1 作文データ

「児童・生徒作文コーパス」（ 宮城・今田 2015）を使用する。このコーパスは小学校 1 年生から中学校 3 年生までの作文を電子化した係り受け構造タグ付きコーパスであり、自然言語の特性、および学齢による構造の変化を観察するために適している。コーパスの規模を 表 1 に示す。

**表 1.** 「児童・生徒作文コーパス」の規模。

学年	作文数	文数	文節数
1	405	2896	16788
2	407	4005	25793
3	402	5126	36016
4	433	6190	46429
5	448	6524	49937
6	455	6332	49784
7	930	15030	121581
8	932	16010	125108
9	914	15398	127099
Total	5326	77511	598535

このコーパスを使用して、文長および係り受け距離の分布を調べる。文長については1文あたりの文節数をデータとする。係り受け距離については1文あたりの係り受け距離の平均 (MDD; Liu 2008) をデータとする。いずれも裾が長い分布を示すため、対数正規分布およびガンマ分布によるフィッティングを行い、当てはまりのよさを調べる。さらに文長については学年を固定効果、作文 ID をランダム効果とする一般化線形混合モデル分析、MDD については文長、学年および交互作用を固定効果、作文 ID をランダム効果とする一般化線形混合モデル分析を行い、文長、MDD、学年の関係を分析する。

### 2.2 ランダムデータ

作文データの自然言語としての特性を明らかにするために、2 種類のランダムデータを対照データとして分析する。文長の分析においては、作文データの文節をランダムに並べ替えた文節シャッフル列を用いる。元データにおける文末文節をシャッフル列でも文末と見なし、ランダムな文節列における文長の分布を調べる。ランダムデータにおける文長は幾何分布に従うことが予測されるため、文長についてはデータの分布が幾何分布に従うことのみ確認する。

MDD の分析においては、文節数 n の文における可能な全ての係り受け構造を列挙した係り受けパターン集合を作成し ^2^、可能な全ての構造が等確率で生起する場合を仮定して、MDD の分布を調べる。可能な係り受け構造のパターン数はカタラン数で計算できるが、文節数に対して指数的に増大するため、文節数 2 から 10 の範囲まで作成する ^3^。MDD は裾が長い分布を示すため、対数正規分布およびガンマ分布との当てはまりを確認し、さらに一般化線形モデルで MDD と文長の関係を分析する。作文データと異なり著者による個体差を伴わないので、混合モデルによる分析は行わない。

### 2.3 実験環境

全ての実験は R 言語を用いて行い、分布のフィッティングは MASS::fitdistr 関数、一般化線形モデル分析は stats::glm 関数、一般化線形混合モデル分析は lme4::glmer 関数を使用した。主なソフトウェアのバージョンを 表 2 に示す。

**表 2.** 実験環境。

ソフトウェア	バージョン
R	4.2.1 (x86_64-apple-darwin17.0)
stats	4.2.1 (attached base packages)
MASS	7.3-58.1
lme4	1.1-31

## 3. 文の長さの分布

### 3.1 ランダムデータ

文がランダムに生成された場合、文長（文節数）の分布はどのようになるだろうか。本節では、作文データの文節をランダムにシャッフルした文節シャッフル列を用いて、文長の分布を調べる。元のデータで文末だった文節を、文節シャッフル列においても文末とする。文節シャッフル列は、長さ 598535 の文節列である。このうち文末文節の数は 77512 である。従って、文末文節は 77512/598535=0.1295029 程度の確率でランダムに生起することになる。

このような文節列において、文長の分布は幾何分布に従うと予想される。幾何分布は、確率 p で成功する事象が次に成功するまでの失敗数の分布とされる。確率 p で文末文節が生起する文節シャッフル列においては、次に文末文節が生起するまでの非文末文節の数x（=文長 *n*-1）は次の幾何分布に従うと考えられる。

$$P\left(X=x\right)=p{\left(1-p\right)}^{x}$$

図 1 は、文節シャッフル列における文長のヒストグラムと、 p=0.1295029 の幾何分布曲線を重ね合わせたものである。ヒストグラムに曲線がよく当てはまることが確認できる。以上から、ランダムな文節列においては、文長は幾何分布に従うと考えることができる。

**図 1.**
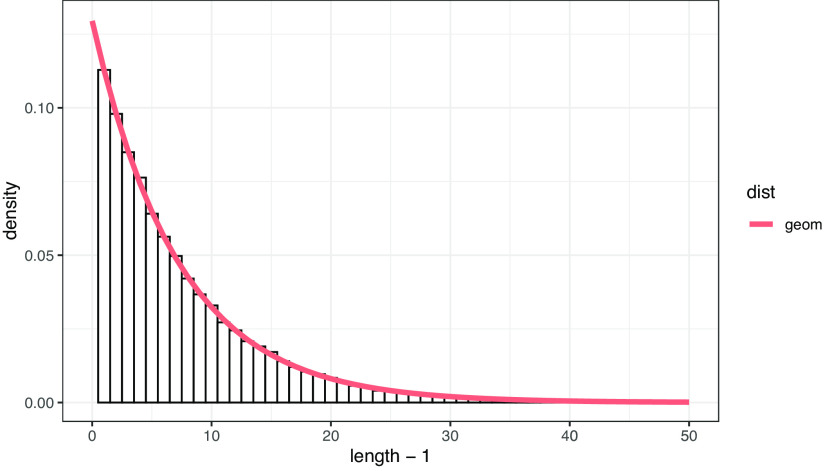
ランダムデータにおける文長（文節数）の分布。 曲線は幾何分布による予測曲線。分布の母数は文末文節の出現確率に基づく。

### 3.2 作文データ

文節シャッフル列における文長の分布が幾何分布に従うのに対して、実際の作文データにおける文長の分布は幾何分布とは異なり、対数正規分布ないしガンマ分布に類似する裾の長い分布を示す。 図 2 は、作文データにおける文長のヒストグラムと、フィッティングによって推定した対数正規分布およびガンマ分布の確率密度曲線を重ね合わせたものである。対数尤度はガンマ分布が -213659.0、対数正規分布が -213695.8 で、ガンマ分布の方がわずかに当てはまりがよい。

**図 2.**
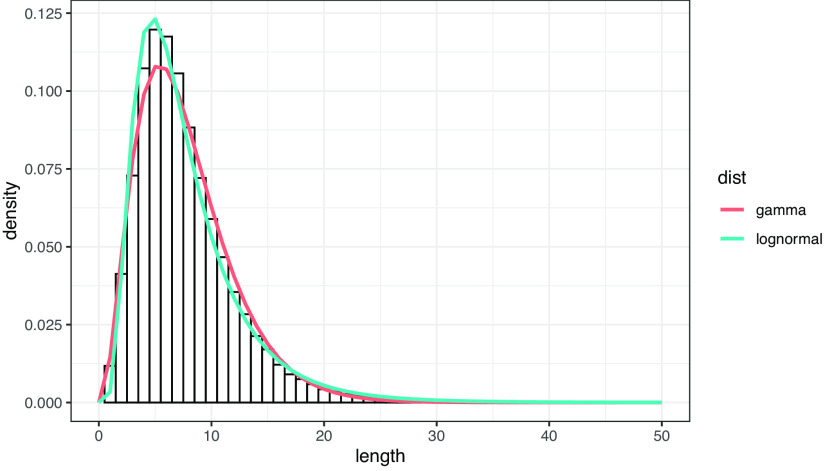
作文データにおける文長（文節数）の分布。 曲線はガンマ分布および対数正規分布によるフィッティング。フィッティングは R 言語の MASS::fitdistr 関数による。

対数正規分布とガンマ分布のどちらの当てはまりがよいかは、学年によって異なる。 図 3 は、学年ごとに 2 種類の分布のフィッティングを行い、対数尤度比 (LLR) をプロットしたものである。LLR が 0 より大きいとき、ガンマ分布の方が当てはまりがよい。学年別に見ると、小 1 から小 6 までは対数正規分布の当てはまりがよく、中 1 から中 3 まではガンマ分布の当てはまりがよい。このデータは、一文あたりの文節数の分布は、年齢が低いときには対数正規分布になるが、年齢が上がるに従ってガンマ分布に接近することを示唆する。

**図 3.**
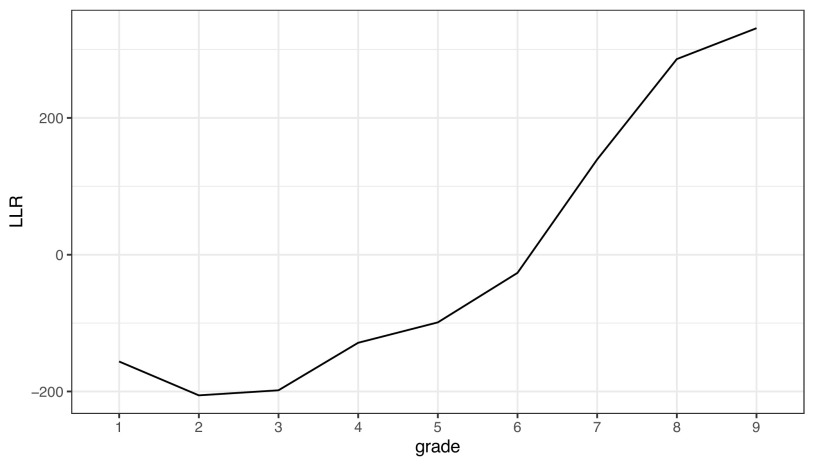
作文データにおける文長（文節数）の対数尤度比。 0 より大きいときはガンマ分布の当てはまりがよく、0 より小さいときは対数正規分布の当てはまりがよい。

従って全ての学年を単一のモデルで分析することには限界があるが、ここでは次の 2 つのモデルで一般化線形混合モデル分析を試みる。

glmer(length ~ grade + (1|document_id), family = Gamma(link = “identity”))

glmer(length ~ grade + (1|document_id), family = gaussian(link = “log”))

どちらのモデルも被説明変数を文節数 (length)、固定効果を学年 (grade)、作文ID (document_id) を変量効果としたモデルである。第 1 のモデルは文長の分布をガンマ分布と仮定し、第 2 のモデルは文長の分布を対数正規分布と仮定している。学年はカテゴリ変数としている。作文 ID は、著者の個体差による効果を説明するためにランダム効果として加えている。結果を 表 3 に示す。決定係数や AIC を見ると、全体としてはガンマ分布モデルの方が当てはまりはよいようである。

**表 3.** 作文データにおける文長（文節数）の一般化線形混合モデル分析。 R 言語の lme4::glmer 関数による。

	Gamma model	Lognormal model
(Intercept)	6.148 (0.117) ***	1.806 (0.015) ***
grade2	0.616 (0.165) ***	0.082 (0.020) ***
grade3	1.321 (0.165) ***	0.174 (0.020) ***
grade4	1.695 (0.162) ***	0.224 (0.020) ***
grade5	1.793 (0.159) ***	0.244 (0.020) ***
grade6	2.089 (0.161) ***	0.293 (0.020) ***
grade7	2.399 (0.140) ***	0.315 (0.017) ***
grade8	2.090 (0.139) ***	0.277 (0.017) ***
grade9	2.568 (0.140) ***	0.336 (0.017) ***
SD (Intercept document_id)	1.430	1.118
SD (Observations)	0.531	4.105
Num.Obs.	77511	77511
R2 Marg.	0.143	0.000
R2 Cond.	0.896	0.069
AIC	416392.4	465568.8
BIC	416494.2	465670.7
ICC	0.9	0.1
RMSE	4.22	4.10

*** p < 0.001.

ガンマ分布モデルについて、学年が文長に及ぼす効果の予測値を 図 4 に示す。学年が上がるに従って文長が長くなることが確認できるが、一方で学年が上がるほど文長の増加速度は緩やかになる。

**図 4.**
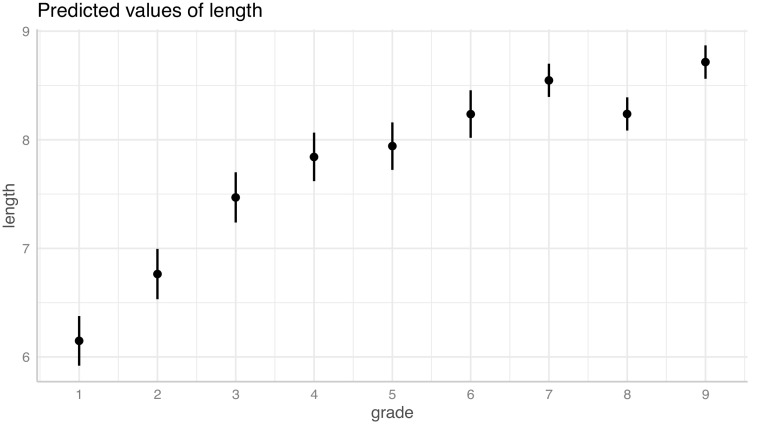
一般化線形混合モデルによる文長（文節数）の予測値。 髯は 95%信頼区間。R 言語の lme4::glmer 関数による解析結果を ggeffects::ggpredict 関数で可視化した。

## 4. 係り受け距離平均の分布

### 4.1 係り受け距離平均

前節では文長（文節数）の分布について検討したが、同じ文節数の文でも係り受け構造には多様なパターンがあり、それによって文処理のための認知的負荷が異なることが考えられる。ここでは係り受け距離が文の複雑さの一側面を表すものと仮定して、ランダムな構造や自然言語の構造において係り受け距離がどのような分布を示すか検討する。

なお、本稿では日本語の規範的な係り受け構造のみを検討対象とする。規範的な係り受け構造においては、文末以外の全ての文節は自分より後ろの文節に係り、文末の文節は係り先を持たない。1 つの文節は、最大で 1 つしか係り先を持たない（従って、1 文に含まれる係り受けの数は文末を除いた文節数と同じであり、文節数 *n* の文における係り受けの数は *n*-1 である）。また、係り受けは交差しない（例えば第 1 文節が第 3 文節に係り、第 2 文節が第4文節に係るというような交差はしない）。実際には語順の入れ替えによって後ろから前への係り受けが発生したり、係り受けが交差したりすることはあり得るが、本稿では検討しない。

文単位で係り受け距離を数値化するために、係り受け距離平均 (MDD, Mean of Dependency Distances) を用いる。MDD は、ある文に含まれる全ての係り受けの距離の平均である。ある文に含まれる全ての係り受けの距離の和を SDD とすると、係り受けの数は文節数 *n*-1 なので、MDD=SDD/( *n*-1) になる。例として文節数4の文を考えると、可能な構造は 5 通りあり、それぞれの SDDとMDD は 図 5 の通りである。

**図 5.**
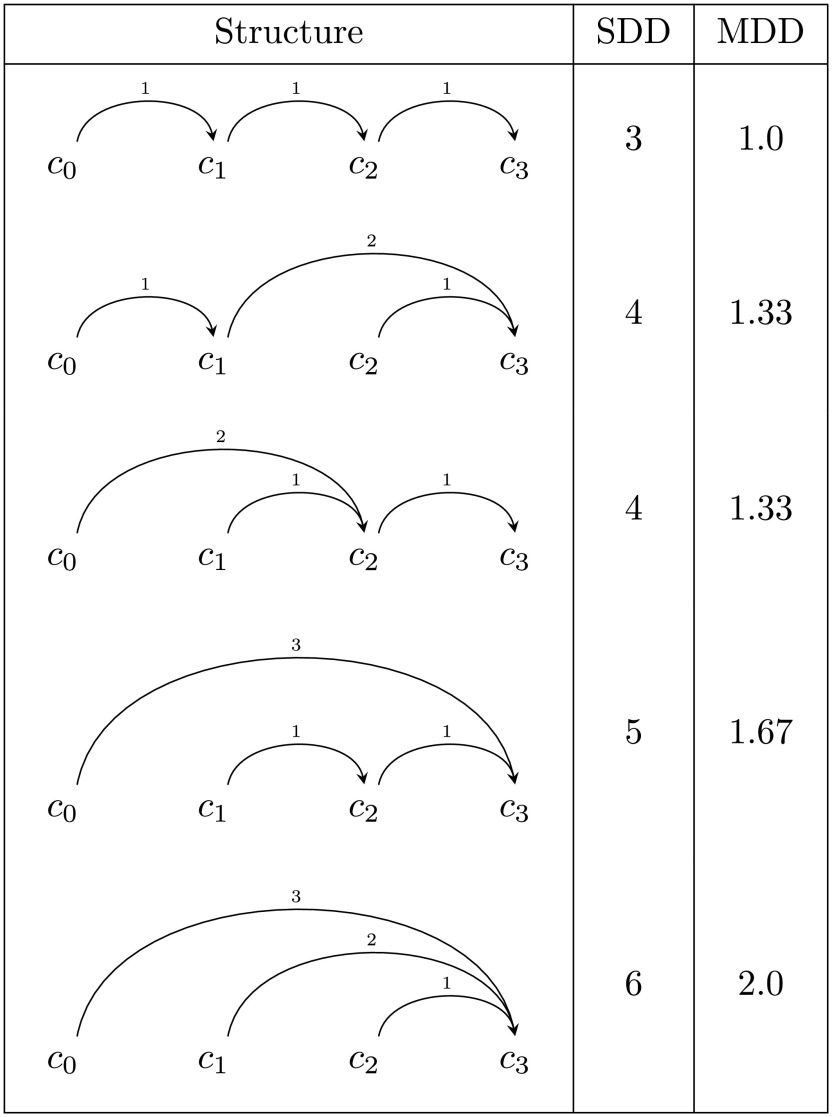
文節数 4 の文における係り受けパターン。 SDD は係り受け距離和、MDD は係り受け距離平均。本稿では日本語係り受け文法の慣例に従って前の文節から後ろの文節にエッジを引くが、依存構造の研究では主辞 (governor) から依存要素 (dependant) にエッジを引くことが一般的であり、矢印の方向が逆になることに注意されたい。

文節数 *n* の文における可能な構造の数はカタラン数 $C_{n-1}$ で計算できる。カタラン数 $C_{n}$ は次の式で定義される。文節数 4 の場合、可能な構造数は $C_{3}=5$ である。

$$C_{n}=\frac{\left(2n\right)!}{\left(n+1\right)!n!}$$

MDD が最小になるのは全ての文節が直後の文節に係る場合であり、最大になるのは全ての文節が文末文節に係る場合である。文節数 n の文における可能な全ての構造の MDD の集合を ${\mathrm{MDD}}_{n}$ とすると、 ${\mathrm{MDD}}_{n}$ の最小値と最大値はそれぞれ次の値を取る。

$$\min\left({\mathrm{MDD}}_{n}\right)=1$$

$$\max\left({\mathrm{MDD}}_{n}\right)=\frac{n}{2}$$

### 4.2 ランダム構造

可能な全ての構造が一様の確率で生起する場合の MDD の分布はどのようになるだろうか。可能な全ての構造を列挙した係り受けパターン集合を用いて MDD の分布を確認することにしよう。例として文節数 10 の文を考えると、可能な構造の数は $C_{9}=4862$ である。これらの構造における MDD の最小値は 1、最大値は 5 である。ヒストグラムを書くと、MDD は裾が長い分布を示し、この分布は対数正規分布によく当てはまる。 図 6 は、ヒストグラムとフィッティングにより推定した対数正規分布およびガンマ分布の曲線を重ね合わせたものである。対数尤度は対数正規分布が -4573.228、ガンマ分布が -4565.008 で、対数正規分布の方がよく当てはまる。

**図 6.**
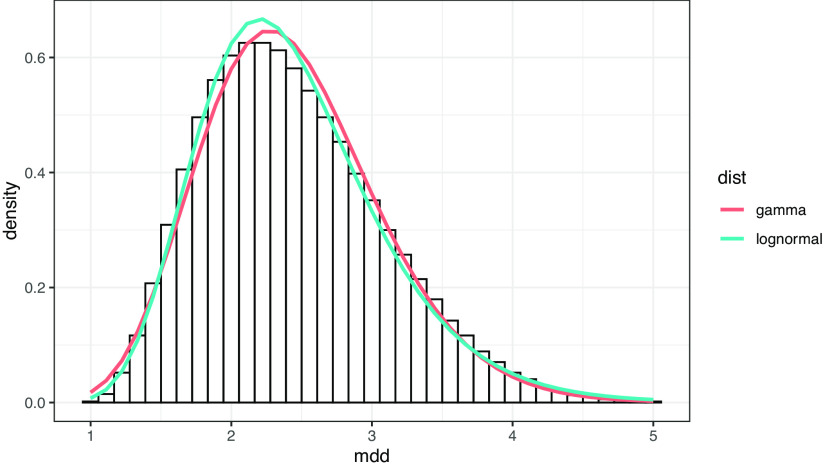
ランダムデータにおける MDD の分布。 曲線はガンマ分布および対数正規分布によるフィッティング。フィッティングはR言語の MASS::fitdistr 関数による。

文節数 *n* が変化すると、MDD の分布はどのように変化するだろうか。 図 7 は文節数に伴うガンマ分布と対数正規分布の LLR の推移と、 $\mathrm{MD}D_{n}$ の平均の推移をプロットしたものである。LLR の推移を見ると、文が長くなるほど対数正規分布の方がよく当てはまることが確認できる。また、文節数 n と $\mathrm{MD}D_{n}$ の平均の推移を両対数グラフにプロットすると、ほぼ線形に分布することが確認できる。すなわち文長と $\mathrm{MD}D_{n}$ の平均は冪関係にある（あるいは文長の対数に対して $\mathrm{MD}D_{n}$ の平均は指数的に増大する）と考えられる。

**図 7.**
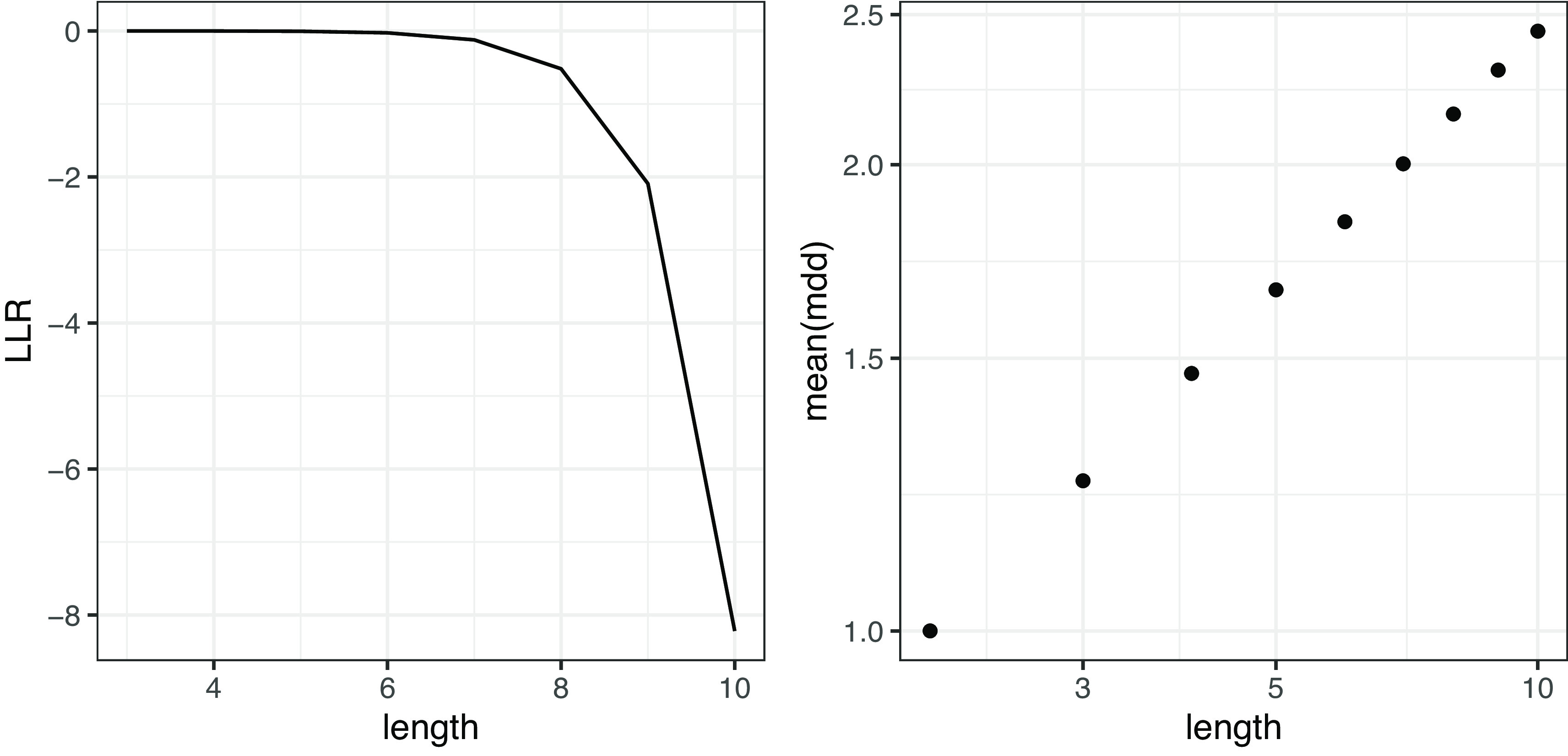
ランダムデータにおける MDD の対数尤度比（左図）と平均値（右図）。 対数尤度比が 0 より大きいときはガンマ分布の当てはまりがよく、0 より小さいときは対数正規分布の当てはまりがよい。右図は両対数グラフ。

そこで、次のモデル式で一般化線形モデル分析を試みる。このモデルは、被説明変数を mdd、説明変数を文節数 length の対数、mdd の分布を対数正規分布とし、mdd の期待値を対数変換すると log (length) と線形関係になると仮定している。

glm(mdd~log(length), family=gaussian(link=”log”))

結果を 表 4 に示す。構築されたモデルによる予測値は、実測による $\mathrm{MD}D_{n}$ の平均の分布とほぼ一致する。以上から、ランダムデータにおける MDD の分布は対数正規分布に従い、その平均は文節数の対数に対して指数的に増大すると考えられる。

**表 4.** ランダムデータにおける MDD の一般化線形モデル分析。 R 言語の stats::glm 関数による。

	Lognormal model
(Intercept)	-0.383 (0.087) ***
log (length)	0.554 (0.039) ***
Num.Obs.	6917
R2	0.034
AIC	13114.4
BIC	13134.9
Log.Lik.	-6554.195
F	205.794
RMSE	0.62

*** p < 0.001.

ところで、モデルの切片 -0.383 は、係数 0.554 に −log2 を乗じた値とほぼ一致する ^4^。従って、可能な全ての構造がランダムに生起した場合の $\mathrm{MD}D_{n}$ の平均は、おおよそ次の式に従う。

$$\text{mean}\left(\mathrm{MD}D_{n}\right)={\left(\frac{n}{2}\right)}^{0.554}$$

### 4.3 作文データ

作文データにおいても、MDD は裾の長い分布を示す。しかしランダムデータと同様に対数正規分布によく当てはまるかは自明ではない。 図 8 は、作文データから文節数 10 の文のみ抽出し、MDD の分布をヒストグラムにしたもので、曲線は対数正規分布およびガンマ分布でフィッティングした予測曲線である。対数尤度は対数正規分布が -3389.641、ガンマ分布が -3374.439 で、ガンマ分布の方がよく当てはまる。

**図 8.**
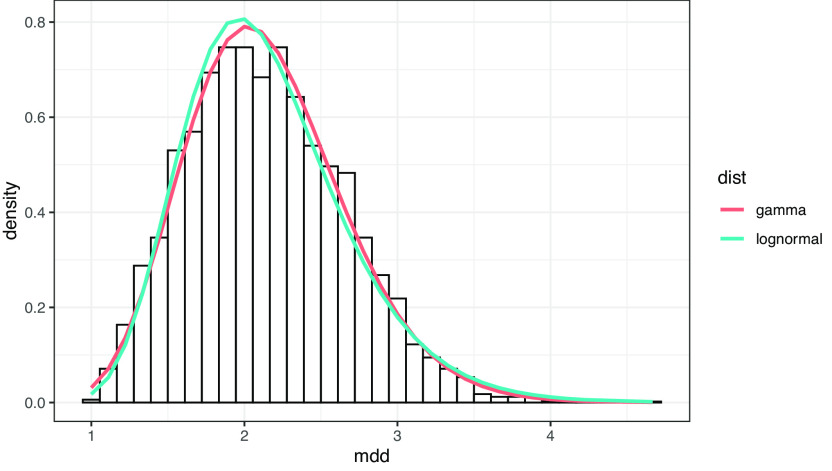
作文データにおける MDD の分布。 曲線はガンマ分布および対数正規分布によるフィッティング。フィッティングはR言語の MASS::fitdistr 関数による。

文節数に伴う LLR の推移と MDD の平均の推移を 図 9 に示す。作文データでは、文節数が 20 を超える文の頻度が小さいため、文節数 20 までのデータを示す。LLR の推移を見ると、文が短いときにはガンマ分布の方がよく当てはまるが、文が長くなると両者の差は小さくなる。MDD の平均の推移を見ると、ランダムデータでは文節数と MDD 平均が両対数グラフで線形に分布するのに対して、作文データでは文節数と MDD 平均が片対数グラフで線形に近い分布を示す。すなわち、MDD の平均は文節数の対数に対して線形であると考えられる。

**図 9.**
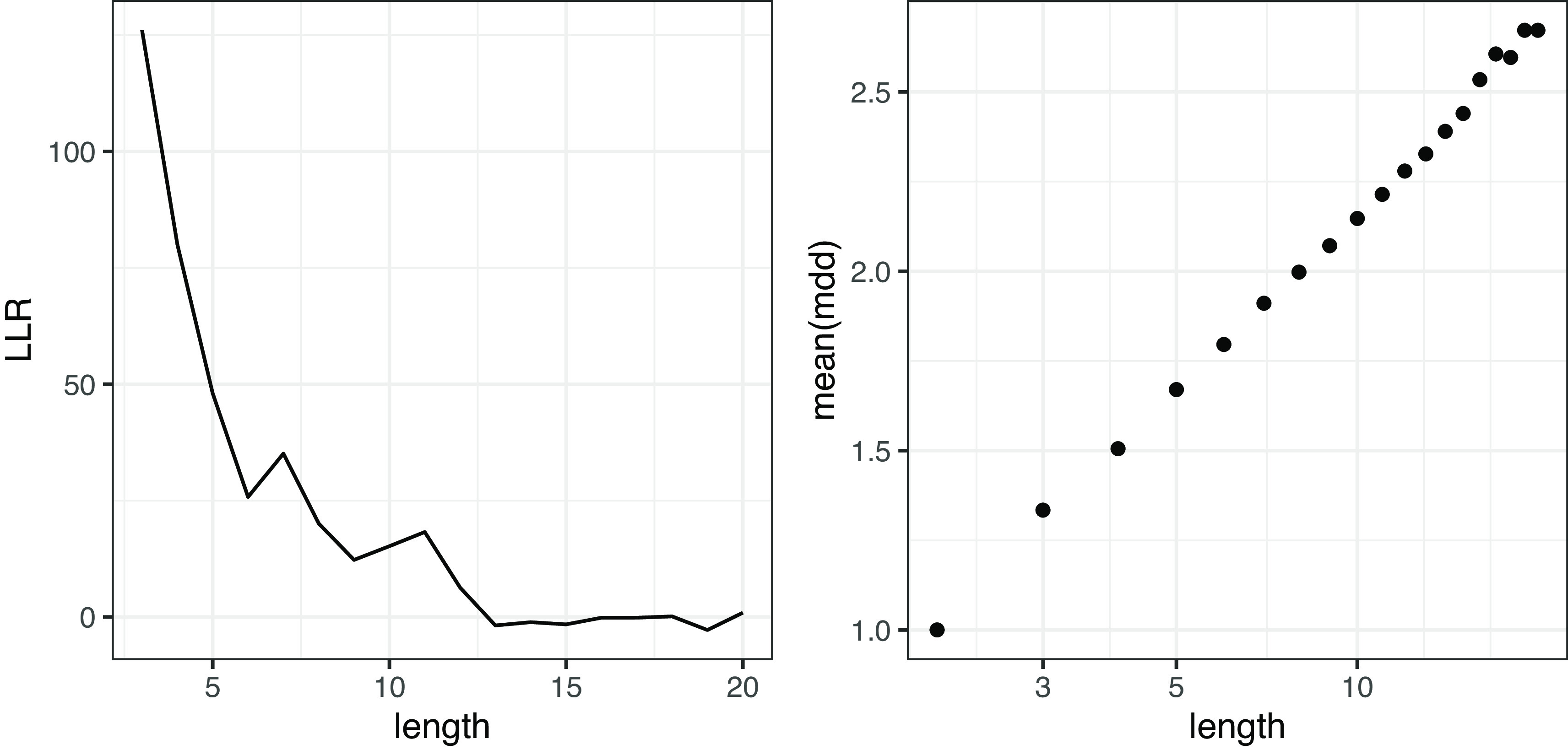
作文データにおける MDD の対数尤度比（左図）と平均値（右図）。 対数尤度比は 0 より大きいときガンマ分布の当てはまりがよく、0 より小さいとき対数正規分布の当てはまりがよい。右図は片対数グラフ。

以上から、2 つのモデルで MDD と文長nの一般化混合モデル分析を試みる。どちらのモデルも被説明変数を MDD、固定効果を文節数 (length) の対数と学年 (grade)、およびそれらの交互作用項、作文 ID (document_id) を変量効果としたモデルである。第 1 のモデルは文長の分布をガンマ分布と仮定し、第 2 のモデルは文長の分布を対数正規分布と仮定している。前者は mdd の期待値がlog (length) に対して線形であることを仮定しており、後者は mdd の期待値の対数がlog (length) に対して線形であることを仮定している。学年はカテゴリ変数である。学年によってlog (length) の係数が変わることが考えられるため、交互作用項をモデルに含めている。作文 ID は、著者の個体差による効果を説明するためにランダム効果として加えている。

glmer(mdd~log(length)*grade+(1|document_id), family=Gamma (link=”identity”))

glmer(mdd~log(length)*grade+(1|document_id), family=gaussian (link=”log”))

結果を 表 5 に示す。決定係数や AIC を見ると、ガンマ分布モデルの方がよく当てはまっている。この結果から、ランダムデータでは MDD が対数正規分布に従うのに対して、作文データでは MDD がガンマ分布に従い、また文長に対する分布の平均の推移も両者では異なると考えることができる。

**表 5.** 作文データにおける MDD の一般化線形混合モデル分析。 R 言語の lme4::glmer 関数による。

	Gamma model	Lognormal model
(Intercept)	0.534 (0.022) ***	-0.153 (0.017) ***
log (length)	0.705 (0.014) ***	0.399 (0.009) ***
grade2	-0.080 (0.030) **	-0.123 (0.023) ***
grade3	-0.098 (0.030) ***	-0.051 (0.022) *
grade4	-0.080 (0.029) **	-0.023 (0.022)
grade5	-0.064 (0.028) *	-0.008 (0.022)
grade6	-0.030 (0.029)	0.005 (0.022)
grade7	0.012 (0.026)	0.065 (0.020) ***
grade8	0.057 (0.025) *	0.077 (0.020) ***
grade9	0.094 (0.026) ***	0.121 (0.020) ***
log (length) × grade2	0.049 (0.018) **	0.067 (0.011) ***
log (length) × grade3	0.067 (0.018) ***	0.033 (0.010) **
log (length) × grade4	0.070 (0.017) ***	0.025 (0.010) *
log (length) × grade5	0.046 (0.017) **	0.009 (0.010)
log (length) × grade6	0.024 (0.017)	0.000 (0.010)
log (length) × grade7	-0.005 (0.015)	-0.033 (0.009) ***
log (length) × grade8	-0.038 (0.015) *	-0.045 (0.009) ***
log (length) × grade9	-0.061 (0.015) ***	-0.068 (0.009) ***
SD (Intercept document_id)	0.085	0.063
SD (Observations)	0.226	0.445
Num.Obs.	76597	76597
R2 Marg.	0.715	0.175
R2 Cond.	0.750	0.191
AIC	81513.2	94971.3
BIC	81698.1	95156.3
ICC	0.1	0.0
RMSE	0.45	0.44

* p < 0.05,

** p < 0.01,

*** p < 0.001.

ガンマ分布モデルの固定効果を見ると、学年の係数は小1から小3まで減少するが、その後は増加する。文長と学年の交互作用項は小学 1 年生から4年生にかけて増加したあと、中学 3 年生まで減少する。このうち、前者は文が短いときに MDD に強く影響し、後者は文が長いときに MDD に強く影響する。固定効果の予測値を見ると、実際にそのようになっていることが確認できる（ 図 10）。

**図 10.**
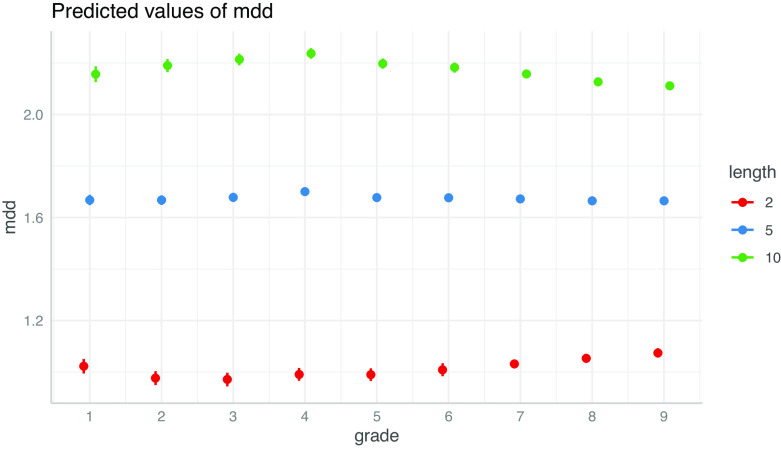
一般化線形混合モデル分析による MDD の予測値（文節数2、5、10のとき）。 髯は 95%信頼区間。R 言語の lme4::glmer 関数による解析結果を ggeffects::ggpredict 関数で可視化した。

## 5. 考察

### 5.1 文長の分布

文長の分布は、ランダム列においては幾何分布に従い、作文データにおいては低学年においては対数正規分布に近いが、学年が上がるに従ってガンマ分布に接近することを確認した。またその平均は学年に従って増加するが、高学年になるに従って増加の仕方が緩やかになる。

ランダム列ににおける文長の分布が幾何分布に従う理由は容易に解釈することができる。ランダム列においては文末文節が一定の確率 p で生起するので、文長の分布は母数 p の幾何分布に従う。これに対して、作文データにおける文長の分布が対数正規分布やガンマ分布に従う理由は自明ではない。 古橋・早川(2012) は京都大学テキストコーパスを用いて文節を単位とする文長の分析を行い、対数正規分布と負の二項分布（ガンマ分布の離散確率分布に相当するもの）では後者の方がよく当てはまることを報告している。また、2 つの分布の生成過程について検討し、対数正規分布の場合は依存構造木が乗算過程的に枝の数を増やしていくプロセスによって生成され、負の二項分布の場合は部分木の長さが幾何分布に従う場合にその加算過程によって生成される可能性を提示しているが、どちらのモデルもデータを説明するには不十分であると結論づけている。

負の二項分布は p と r という2つの母数を持ち、母数 p の幾何分布に従う回数で成功する事象がr回成功するまでの回数の分布として解釈される。文長にあてはめて考えると、部分木の長さが母数 p の幾何分布に従うのであれば、r 個の部分木を持つ文の長さは負の二項分布に従うと考えられる（ガンマ分布と指数分布で考えても基本的には同じである）。だが自然言語の文では部分木の数は常に同じではなく、各部分木の長さも常に同じ母数の確率分布に従うわけではない。文がいくつの節を持つかは文ごとに異なり、節の長さもその種類によって異なる。例えば連体節は短く、並列節は長いといった違いがあり得る。また、著者やテキストの属性によって、使用される節の種類の内訳も異なる。例えば著者の学年が上がるほど連体節の割合は多くなる傾向があるし、話し言葉と書き言葉では後者の方が連体節の割合が多くなるかも知れない。さらに言えば、低学年ほど対数正規分布がよく当てはまり、高学年ほどガンマ分布がよく当てはまるという規則性が見られることは、どちらかの分布が正しいモデルで、偶発的に別の分布の当てはまりがよくなる場合があるというわけではないことを示唆する。しかしいずれにせよ、文節を単位とした場合にも文長の分布が右に歪んだ分布になることは明らかであり、多くの場合においてガンマ分布は（少なくとも正規分布などより）よい近似になると考えられる。

学年を説明変数とする一般化混合モデル分析からは、学年に従って文長が長くなることが確認できる。これは学齢の上昇に伴って言語能力が発達し、より構成要素が多い複雑な文を生成することができるようになることを示していると解釈できる。一方で、文長の増大は学年が上がるほど緩やかになる。直感的には、文は原理的には無限に長くすることができるものの、我々は通常、特に書き言葉においては適切な長さで文を切る方略を用いるので、ある程度の長さの文が書けるようになると、それ以上は文を長くしないものと解釈できる。

### 5.2 係り受け距離平均の分布

MDD の分布は、ランダム構造においては対数正規分布に従い、作文データにおいては概ねガンマ分布に従う。また文が長くなると、ランダム構造の MDD は文長の対数に対して指数的に増加するが、作文データでは線形に増加する。学年は、MDD と文長の線形関係の切片と係数に影響を及ぼすが、切片は学年が上がるほど大きくなり、係数は小学4年まで増加した後は減少する傾向が見られる。

対数正規分布が乗算過程によって生成され、ガンマ分布が加算過程によって生成されることを考えると、前者が指数的、後者が線形的に増大することは自然な帰結かも知れない。しかしながら、なぜ MDD の分布が対数正規分布やガンマ分布に従うかは、文長の分布の場合と同様十分に明らかではない。対数正規分布が生成される過程については、ある程度の手がかりがある。ランダム構造においては、文節数n、係り受け距離和sの可能な構造のパターン数は次の漸化式で計算することができる ^5^。MDD は係り受け距離和を *n*-1で除した値なので、 $D_{n,\left(n-1\right)\mathrm{MDD}}$ とすれば同じ式で分布を計算することができる。本稿はこの式の代数的な解を示すことができないが、これを解くことでランダム構造における MDD の分布のより正確なモデルを得ることができるはずである。

$$\begin{array}{ll} D_{1,0}=1\\ D_{n,s}=\sum_{i=1}^{n-1}\sum_{j=i-1}^{\frac{i\left(i-1\right)}{2}}D_{i,j}D_{n-i,s-i-j} & \left(n>1,n-1\leq s\leq \frac{n\left(n-1\right)}{2}\right)\\ D_{n,s}=0 & \left(\text{otherwise}\right) \end{array}$$

文節数の対数に従った MDD の増加が、ランダム構造では指数的であるのに対して、作文データでは線形であることは、前者よりも後者（すなわち自然言語）の方が MDD の増加が抑制的であることを示している。これは従来よく知られているように、自然言語が長い係り受けを避ける傾向があることの当然の帰結と言える。一方で、作文データの MDD は常にランダム構造の MDD より小さいわけではない。実データの分布（ 図 11）を見ると、文長が 5 文節未満のごく短い文においては、ランダム構造よりも作文データの方が MDD の平均が大きい。

**図 11.**
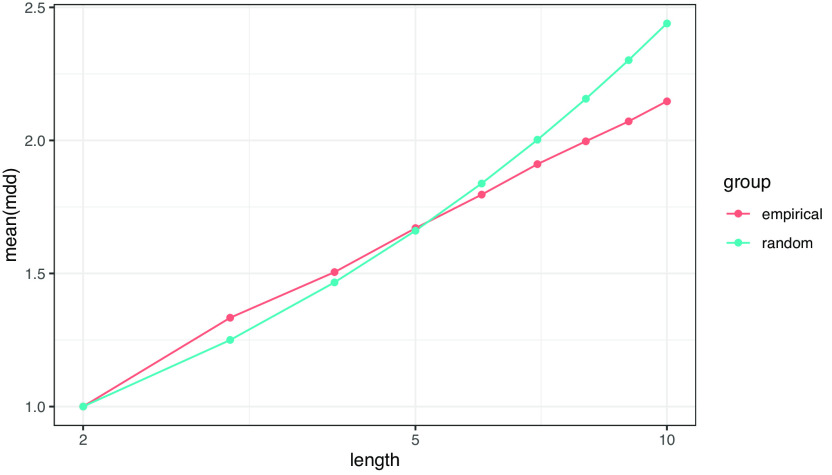
ランダムデータ (random) と作文データ (empirical) における MDD 平均の実測値。片対数グラフ。

文法的な観点から見ると、自然言語における MDD の分布には少なくとも2つの原則が寄与しているように思われる。1 つは節や句の形成に伴う MDD の増大の原則で、例えば動詞句では主語、目的語、副詞などの構成要素が句末の動詞に係ることによって MDD が増大する。もう 1 つは複数の句による階層構造の形成に伴う MDD の最適化の原則で、長い句を前に、短い句を後ろに置くことで長い係り受けをなるべく少なくするように構造が作られる。文節が 4 つ程度までの短い文は階層構造を持たない単文が多く、全ての文節が文末の述語に係る。そのため、ランダム構造よりも MDD が大きくなる傾向が見られる。それより長い文になると文中に階層構造が現れ、最適化の原則によってランダム構造よりも MDD が小さくなる。

2 つの原則は、回帰モデルにおける切片と係数の解釈にも関連する。MDD が log(length) と線形関係にあると仮定すると、MDD は文が短いときには切片の影響を強く受け、文が短いときには log(length) の係数の影響を強く受ける。そのため、切片は句における MDD の増大と強く関連し、係数は階層構造におけるMDD の最適化と強く関連する。同様に、学年の係数は MDD の増大と関連し、学年と文長の交互作用項の係数は MDD の最適化と関連する。一般化線形混合モデル分析の結果では、学年の係数は高学年ほど大きくなり、学年と文長の交互作用項の係数は小学4年まで増加した後は減少する傾向が見られた。前者は学齢の進行に伴って句の構成要素を増やすことで複雑な文を形成する能力が発達することを示唆し、後者はある程度の学齢に到達した後に長距離の係り受けを回避し、構造を最適化する能力が発達することを示唆する。

## 6. まとめ

日本語の文の複雑性について、文長（文節数）と係り受け距離平均（MDD）の観点から分析した。ランダムデータと作文データの比較では、前者の文長が幾何分布、MDD が対数正規分布によく当てはまるのに対して、後者の文長は対数正規分布ないしガンマ分布、MDD はガンマ分布によく当てはまることが確認された。また、ランダムデータにおける MDD の平均が文長の対数に対して指数的に増大するのに対して、作文データにおける MDD の平均は文長の対数に対して線形に増大することが分かった。この結果は、自然言語における係り受け距離の分布は無作為な構造と比べて有意に小さくなるという従来の知見を支持するものだが、本稿の分析からはその差が係数の違いに留まるものではなく、指数的か線形かという関数の特性のレベルで異なることが示された。また、文節数がごく小さい文においてはランダムデータよりも作文データの方が係り受け距離平均が大きくなることも分かった。

作文データの分析においては、学年が上がるほど文長が長くなることが確認された。また MDD については、学年による効果は減少した後に増大し、学年と文長の交互作用は増大した後に減少することが分かった。MDD の非単調な変化は、児童における統語構造の構成能力の発達が、構造の複雑化と最適化の 2 つの側面を持つ複雑な過程であることを示唆する。児童はより構成要素の多い複雑な構造を構成する能力を発達させるのと並行して、係り受け距離の短い最適化された構造を構成する能力を発達させているかも知れない。特に前者の能力については、係り受け構造におけるノード次数の分布など、より直接的な証拠の分析が追加で必要だろう。

他の残された課題の 1 つは分布を生成する過程の解明である。特に作文データにおける文長や MDD の分布がなぜランダムデータとは異なるロングテール分布になるかは十分に分かっておらず、そのためガンマ分布の当てはまりのよさも現時点では近似に過ぎないと言わざるを得ない。しかしながら、日本語の文節係り受け構造における文長や MDD の比較的良い近似が確認できたことは応用的研究のために有益である。今後、分布の生成モデルの検討とともに、文章の可読性の分析や文章ジャンルによる文構造の差異の分析への応用を進めたい。

## データ可用性

### 基礎データ

データはすべてオープンサイエンスフレームワークで利用可能です。児童作文における文節数および係り受け距離の分布. https://doi.org/10.17605/OSF.IO/3YAQU ( Imada, 2023).

このプロジェクトには以下の基礎データが含まれています。

• sakubun_chunks.csv（「児童・生徒作文コーパス」ver.1.6 から文節情報のみを抽出したデータです。本文を含むオリジナルのデータは公開されていません。）
• random_chunks.csv（文節数 2 から 10 までの範囲で、可能な係り受け構造を全て列挙したデータです。）

### 拡張データ

解析コードはオープンサイエンスフレームワークより入手可能です。

アーカイブされた解析コード（論文公開時）: https://doi.org/10.17605/OSF.IO/3YAQU

データは、 Creative Commons Zero “no rights reserved” data waiver (CC0 1.0 Public domain dedication) の条件下で利用可能です。

## 同意

本研究では「児童・生徒作文コーパス」から取得した文節係り受け情報をデータとして使用している。このデータは作文本文を含まず、また公開にあたって同意を必要とする著者の個人情報は含まれていない。なお、「児童・生徒作文コーパス」はプロジェクト関係者のみが利用可能な非公開データであり、データ構築の時点で著者の特定に繋がるデータは作文本文中の固有名などを含めて全て被覆されている。

## Footnotes

^1^
ここで引用する論文は「言語資源活用ワークショップ 2021」（2021 年、国立国語研究所コーパス開発センター主催学会）の発表原稿であり、DOI を付与して公開されているが、査読を経ていない。本論文は、同学会で発表された研究の一部を再検討し、内容や分析方法に相当の改変を加えたものであり、発展的研究の成果として位置づけられる。

^2^
別のランダムデータの生成方法として、実際のデータの依存構造を保持したまま構成要素の線形順序をシャッフルしてデータを生成する方法が考えられる。 Futrell *et al.* (2015) はこの方法で、語順をランダムに入れ替えたデータ、枝分かれの方向を固定して語順をランダムに入れ替えたデータなどをベースラインとして検討している。 Ferrer-i-Cancho *et al.* (2022) はある構造 D の語順をランダムに入れ替えたときの最小値 D _min_ と平均 D _rla_ をベースラインとして使用しており、この構造では依存構造の交差も許される。本稿で使用するパターン集合は、枝分かれの方向は固定し（日本語の文節係り受け構造では、規範的には前の文節から後ろの文節に係り受けする）、交差は認めず、実データの構造に依らず可能な全ての構造を生成する。

^3^
本研究では一般化線形モデル分析のためにランダムデータのセットを生成したが、単に MDD の分布を取得するだけであれば漸化式を用いて比較的小さなコストで計算することができる（5.2 節参照）。

^4^

${\mathrm{MDD}}_{n}$ の最小値が 1 、最大値が n/2 であることから、平均も ${\left(n/2\right)}^{x}$ に従うことが考えられる。これを対数化すると xlogn−xlog2 であるため、平均が ${\left(n/2\right)}^{x}$ に従うのであれば切片は係数xに −log2 を乗じた値になると予想され、実際にその値が得られた。

^5^
この漸化式は以下のアルゴリズムに基づく。文節数が 1 の文における可能な構造パターンは1通りのみである。この構造は係り受けを持たないので $D_{1,0}=1$ である。
文節数が 2 以上の文は、文を 2 つの部分構造に分割して考える。2 つの部分構造は、前方の部分構造の末尾文節から後方の部分構造の末尾文節（すなわち文末）への係り受けによって結合されているものとする（二分木型の句構造に置き換えるならば、最上位の句接点で 2 つの部分構造が分割されていると考えられたい）。 2 つの部分構造の長さを $n_{1}$ 、 $n_{2}$ とし、SDD を $s_{1}$ 、 $s_{2}$ とする。このとき、文全体の長さは $n=n_{1}+n_{2}$ である。また、文全体の SDD は部分構造の SDD の和に 2 つの部分構造を結合する係り受けの長さを足したものになる。2つの部分構造を結合する係り受けの長さは後方の部分構造の長さ $n_{2}$ と同じなので $s=s_{1}+s_{2}+n_{2}$ である。ここで $n_{2}=i$ 、 $s_{2}=j$ とすると、 $n_{1}=n-i$ 、 $s_{1}=s-i-j$ なので、文全体の可能な構造パターン数は $D_{i,j}\times D_{n-i,s-i-j}$ となる。これを $n_{2}$ および $s_{2}$ が取りうる全ての *i*, *j*について計算し、その総和を求めることで $D_{n,s}$ の値を求めることができる。 *i*の変域は[1, *n*-1]である。 *j*の変域はiに応じて変化し、[ *i*-1, *i* ( *i*-1)/2]である。従って $D_{n,s}=\sum_{i=1}^{n-1}\sum_{j=i-1}^{i\left(i-1\right)/2}D_{i,j}D_{n-i,s-i-j}$ である。
ただし、文節数 *n*の文における *s*の最小値は *n*-1、最大値は *n*( *n*-1)/2である。sがこの範囲を逸脱する構造は存在しないので、その場合には $D_{n,s}=0$ である。
