# Supplementary material for: Distribution of sentence length and dependency distance in children’s compositions: Characteristics of natural language and variations in language development
Source: F1000Res. 2023 Apr 11;12:379. [Article in Japanese] [Version 1] doi: 10.12688/f1000research.132383.1 (PMC10291895; doi:10.12688/f1000research.132383.1)
Supplement: Peer review history

# Peer review: Reviewer response for version 1

**DOI:** 10.5256/f1000research.145297.r169127

**Published:** 2023-06-23

**Authors:** Saeko Komori, Masatoshi Sugiura

**Affiliations:** 1 Nagoya University, Nagoya, Japan; 2 Chubu University, Kasugai, Japan

日本語を母語とする「児童・生徒作文コーパス」データにおける文長（文中の文節数）および係り受け距離平均（MDD）の分布について、小学校１年生から中学３年生までの学年間の変化を、一般化線形混合モデルで分析している。実際の作文データと作文データ内の文節をランダムにしたデータとを比較することにより、自然言語に固有の特性を明らかにしようとしている点に特徴がある。

 自然言語では文長およびMDDの分布がいずれもガンマ分布もしくは対数正規分布に当てはまることが観察されている。どちらの分布が当てはまりが良いかについて、対数尤度比（LLR）で分析し、文長は低学年は対数正規分布、高学年はガンマ分布に従い、MDDは概ねガンマ分布に従うことが観察された。

 自然言語の場合、MDDは、文長の対数と比例関係にあること、そして、文長と学年の交互作用があり、１年生から４年生までは増加するがそれ以降は減少する傾向が見られたと報告されている。文長自体も、学年とともに増加傾向にあるが高学年になると増加が緩やかになると報告されている。こうした分析から、文長もMDDも低学年の間は増加傾向が見られるが、高学年になるにつれ、MDDは文長と学年の交互作用のため必ずしも常に増加傾向にあるとは言えないと言えるであろう。

 こうした点について、言語発達が、統語構造の複雑化と最適化の二つの側面を持つという考察をしているが、これは卓見であるといえよう。人の認知処理能力（ワーキングメモリー容量等）に上限があるため、MDDは必ずしも上がり続けないというLiu (2008)の研究との関連性も議論できるのではないかと思われる。

 内容が高度で複雑であるため理解が難しいという面もあるが、もう少しわかりやすく書くこともできたのではないかと思われる点もある。例えば、「前者は」「後者は」という指示詞が複数個所で使われているが、話が複雑なため、それぞれが具体的に何を指すのかがわかりづらい。「前者」「後者」の内容を明示的に書いた方が読みやすくなると思われるため、この点は改訂していただきたい。

 細かい点であるが、確認していただきたい点がある。11ページ3行目と4行目に「文長の分布」とあるが、この二か所はいずれも「文長」ではなく「MDD」のことではないかと思われる。もう一点、14ページ第3段落2行目に「文が短いとき」が2回出てくるが、2つ目は「文が長いとき」のことではないかと思われる。確認をし、間違っていれば訂正をしていただきたいし、間違っていないとしたら、間違って読まれないように表現を工夫していただきたい。

 本論文は文長とMDD両方の分布の言語発達に伴う推移をGLMMを使って分析した論文として大変意義のある論文であるといえる。GLMMによる文長の学年進行に伴う予測値については図４で示されているが、MDDの学年進行に伴う予測値の図が掲載されていない。この論文にとってMDDのグラフは画竜点睛であり、是非とも掲載するよう改訂していただきたい。

 また、これは今後の課題として位置づけてもよいが、トピックの影響についての議論、もしくは説明もあった方が望ましい。一般的に言って、トピックの違いにより作文データは影響を受ける。その影響について今回の分析ではどのように対処したのか、もしくは、何らかの理由で、しなかったのかという点の説明があった方が論文としての説得力を増すと思われる。また、データにつていて、小学校から中学校までのデータを連続した9年間として扱っているが、同じ小学校内であれば、1年生から6年生までの横断的データだとしてもほぼ等質であろうと想定されるが、小学校と中学校は通例、学校が違うので、それだけでも何らかの違いを生じさせることにならないかと懸念される。例えば、表1で作文データの規模が示されているが、小学校と中学校の間は連続的な変化ではなく、中学校の方が桁違いに多い。また、図３で文長の対数尤度比の変化が示されており、グラフとしては折れ線で連続しているかのように見えるが、小学校側はマイナスで中学校側がプラスで、小学校と中学校の境目でマイナスとプラスが逆転していると解釈される可能性がある。こうした、トピックの影響と小学校・中学校のデータの連続性という二点について説明があった方が望ましい。

 「児童・生徒作文コーパス」が、非公開データであるという点は理解できるが、「観察するために適している」（p3. 「2.1 作文データ」第一段落）と判断する根拠として、データとしてのコーパスの特徴（上記のトピック等）に関する具体的な情報をここで提示しておいた方が良いと思われる。

（該当する場合は要回答）統計分析および解釈は適切ですか。

一部該当

本研究は明確かつ正確に提示されたものであり、最新の文献を引用していますか。

一部該当

方法と分析について第三者による再現が可能となるよう十分な詳細が提示されていますか。

一部該当

結果の基礎となるソースデータはすべて入手可能で再現性を十全に保証していますか。

はい

結論は結果により妥当な裏付けを得ていますか。

はい

研究設計は適切で学術的価値がありますか。

はい

Reviewer Expertise:

第二言語習得論、学習者コーパス

We confirm that we have read this submission and believe that we have an appropriate level of expertise to confirm that it is of an acceptable scientific standard.

# Peer review: Reviewer response for version 1

**DOI:** 10.5256/f1000research.145297.r169125

**Published:** 2023-05-19

**Authors:** Teiko Arai

**Affiliations:** 1 Tohoku University, Sendai, Japan

　本稿は、日本語の文章の複雑性について、文長と係り受け距離平均(MDD)の観点から分析している。分析に際しては、ランダムデータと児童作文データを比較し、学年による分布の変化を検討する方法がとられている。その結果、ランダムデータと作文データを比較すると、作文データの文長とMDDの分布がランダムデータとは異なるロングテール分布になること、そして作文データでは学年が上がるほど文長が長くなり、MDDについては、学年による効果は減少した後に増大し、学年と文長の交互作用は増大した後に減少することが報告されている。

 　児童の言語発達という難しい課題に対し、義務教育段階の9学年の質の良い作文コーパスが構築されたことの学術的有用性と教育的意義は極めて大きい。学習者の著作権・プライバシー等の事情から、作文コーパスの数は現状多いとは言えず、その作文コーパスを使用した研究事例は未だ少なく、学習者の作文能力の発達過程の解明が待たれる。例えば本論文では、学年が上がるにつれ文長が長くなるが、その増加速度は緩やかになることが示唆されているが、学年に伴う変化の解釈の確定には未だ多面的な分析が必要になるだろう。

 　表1に示されるコーパス規模のデータから、小学校1年生の1作文あたりの平均文節数が約40であるのに対し、中学校3年生ではおよそ140にまで増加していることが分かる。一方で、これが作文課題や教授（指示等）、作業時間などの影響を受けているのかどうかは論文で説明されていないため、例えば課題が異なる作文群について、文長やMDDを一括して評価することについて懸念がある。この点について明確な説明があればありがたい。また、MDDの結果に関しては、これを児童の言語能力の発達における文構造の複雑化と最適化の2つの側面を持つものとする解釈は、直感的に有望であるが、学習者の追加的調査等の今後の分析によってこの解釈が裏付けられることが期待される。

 　本稿は、児童の構文に関する言語能力に焦点を当てた日本語の計量言語分析の研究事例であり、また、単純な学年（年齢）による発達過程の予想の困難を示唆する結果として評価できる。

（該当する場合は要回答）統計分析および解釈は適切ですか。

一部該当

本研究は明確かつ正確に提示されたものであり、最新の文献を引用していますか。

一部該当

方法と分析について第三者による再現が可能となるよう十分な詳細が提示されていますか。

一部該当

結果の基礎となるソースデータはすべて入手可能で再現性を十全に保証していますか。

一部該当

結論は結果により妥当な裏付けを得ていますか。

一部該当

研究設計は適切で学術的価値がありますか。

一部該当

Reviewer Expertise:

人文情報学

I confirm that I have read this submission and believe that I have an appropriate level of expertise to confirm that it is of an acceptable scientific standard.
